# Protein-dependent membrane remodeling in mitochondrial morphology and clathrin-mediated endocytosis

**DOI:** 10.1007/s00249-021-01501-z

**Published:** 2021-02-02

**Authors:** Daryna Tarasenko, Michael Meinecke

**Affiliations:** 1 Department of Cellular Biochemistry, University Medical Center Göttingen, Humboldtallee 23, 37073 Göttingen, Germany; 2 Göttinger Zentrum für Molekulare Biowissenschaften – GZMB, 37077 Göttingen, Germany

**Keywords:** Membrane dynamics, Mitochondria, Clathrin-mediated endocytosis, Mitochondrial morphology, Mitochondrial ultrastructure, Membrane curvature

## Abstract

Cellular membranes can adopt a plethora of complex and beautiful shapes, most of which are believed to have evolved for a particular physiological reason. The closely entangled relationship between membrane morphology and cellular physiology is strikingly seen in membrane trafficking pathways. During clathrin-mediated endocytosis, for example, over the course of a minute, a patch of the more or less flat plasma membrane is remodeled into a highly curved clathrin-coated vesicle. Such vesicles are internalized by the cell to degrade or recycle plasma membrane receptors or to take up extracellular ligands. Other, steadier, membrane morphologies can be observed in organellar membranes like the endoplasmic reticulum or mitochondria. In the case of mitochondria, which are double membrane-bound, ubiquitous organelles of eukaryotic cells, especially the mitochondrial inner membrane displays an intricated ultrastructure. It is highly folded and consequently has a much larger surface than the mitochondrial outer membrane. It can adopt different shapes in response to cellular demands and changes of the inner membrane morphology often accompany severe diseases, including neurodegenerative- and metabolic diseases and cancer. In recent years, progress was made in the identification of molecules that are important for the aforementioned membrane remodeling events. In this review, we will sum up recent results and discuss the main players of membrane remodeling processes that lead to the mitochondrial inner membrane ultrastructure and in clathrin-mediated endocytosis. We will compare differences and similarities between the molecular mechanisms that peripheral and integral membrane proteins use to deform membranes.

## Clathrin-mediated endocytosis

Cells of many higher eukaryotic organisms have developed mechanisms to take up nutrients, cell surface receptors, and other molecules. The arguably best studied uptake process is called clathrin-mediated endocytosis (CME) (Doherty and McMahon 2009; Saheki and De Camilli 2012; Kirchhausen et al. 2014). It is an essential process of eukaryotic cells, fundamental to signal transduction, neurotransmission, and the regulation of many other activities at the plasma membrane. Here, 40 different proteins come together on the cytosolic side of the plasma membrane in highly orchestrated manner, to sort and cluster cargo molecules and to recruit adaptor and scaffold proteins to form a clathrin-coated pit. During pit maturation, more and more molecules are recruited and the pit grows into a clathrin-coated vesicle that is, in a final step, pinched off the plasma membrane (Slepnev and De Camilli 2000). After internalization, the vesicle gets stripped off clathrin and is either recycled to fuse with the plasma membrane or becomes part of the endosomal pathway. CME became a treasure trove for molecules with the ability to shape biological membranes (McMahon and Gallop 2005; Haucke and Kozlov 2018). A number of proteins involved in CME were found to be transiently attached, peripheral membrane proteins that sense and induce membrane curvature. Taking the highly dynamic membrane remodeling process into account that happens during clathrin-coated vesicle formation, it is conceivable that membrane shaping proteins act on each stage of CME from nucleation over cargo selection and coat assembly to the scission reaction (Taylor et al. 2011).

### Molecular mechanisms of membrane deformation in clathrin-mediated endocytosis

**Fig. 1:**
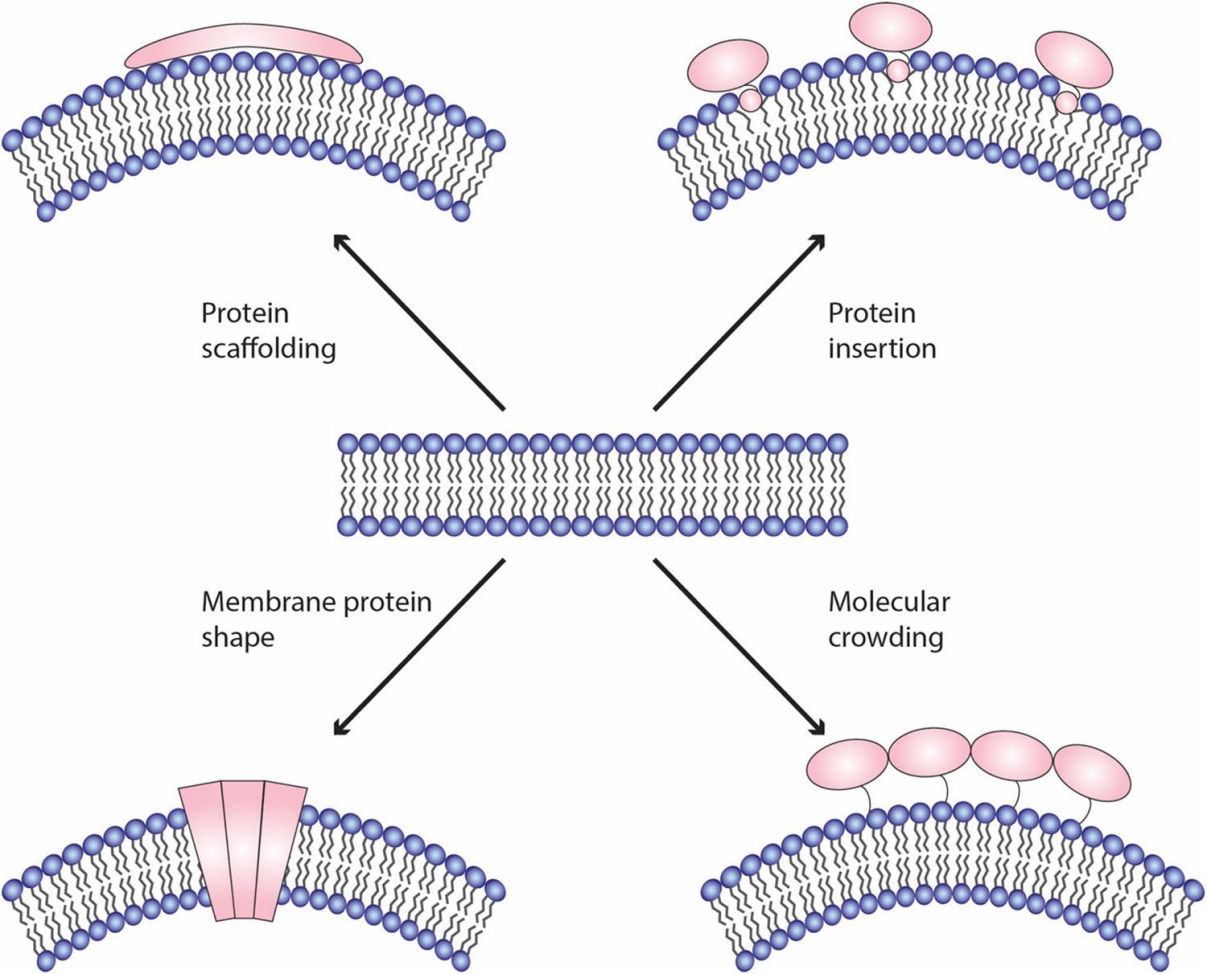
Mechanisms of membrane remodeling. Lipid bilayers can be deformed by inducing positive or negative membrane curvature. Exemplified are four molecular mechanisms by which proteins can remodel membranes

Over the last 20 years, different molecular mechanisms how proteins and lipids can shape biological membranes were described. To study the biochemical and biophysical details of protein-dependent membrane deformation, several model membrane systems were developed (Baumgart et al. 2011). One of the best studied membrane remodeling protein modules is the Bin/Amphiphysin/Rvs (BAR)-domains (Frost et al. 2009; Daumke et al. 2014). BAR domain superfamily containing proteins are found in various membrane trafficking pathways including CME. They transiently interact with membranes and are, in many cases, able to deform membranes. It was early on observed that the addition of purified BAR domains to large unilamellar vesicles (LUVs) leads to broad morphology changes converting round liposomes into elongated tubular structures (Takei et al. 1999; Farsad et al. 2001; Peter et al. 2004) as observed by electron microscopy (EM) on negative stained samples. In vitro membrane binding assays can employ co-sedimentation of protein and LUVs (Ford et al. 2002), co-floatation gradient centrifugations of proteins mixed with LUVs (Barbot et al. 2015) or fluorescent microscopy of giant unilamellar vesicles (GUVs) using labeled proteins (Wollert and Hurley 2010; Meinecke et al. 2013). Several BAR domain-containing proteins act as rigid protein scaffolds that impose their curvature on the underlying membrane while recruiting other proteins involved in CME (Takei et al. 1999; Meinecke et al. 2013). Helical patterns of this protein scaffolds could be visualized using cryo-EM (Frost et al. 2008). BAR domains consist of crescent shape α-helical coiled coil dimers. They bind, in the case of CME, with their concave surface to the negatively charged inner monolayer of the plasma membrane (Peter et al. 2004; Gallop et al. 2006). Here, they stabilize existing membrane curvatures and/or induce curvature that geometrically corresponds to their membrane binding interface. Hence, BAR domains generate defined curvatures and form, in many cases, rigid scaffolds on these membrane regions. The BAR domain superfamily of proteins contains different classes that are characterized by specific geometry and additional domains (McMahon and Gallop 2005; Frost et al. 2009; Daumke et al. 2014), both of which have an influence on membrane binding and curvature induction. N-BAR domains, found, for example, in endophilin and amphiphysin, display an amphipathic helix at their N-terminus. As outlined below, such amphipathic domains have the ability to induce and sense membrane curvature on their own. F-BAR domains of FBP17 or FCHo constitute a shallower arch and thus induce less pronounced curvatures (Shimada et al. 2007; Henne et al. 2007, 2010). I-BAR domains display an inverted geometry where the membrane binding side is convex. While other BAR domains induce positive curvature of different degrees, I-BAR proteins were found to induce negative membrane curvature (Saarikangas et al. 2009). Interestingly, it was shown that CME relies on high-fidelity of the spatiotemporal recruitment and dissociation of different BAR domain proteins (Taylor et al. 2011) (Fig. 1).

Another set of proteins recruited at specific timepoints of CME contain amphipathic domains, mostly amphipathic α-helices. Such amphipathic helices (AH) are found in various proteins and peptides involved in a wide range of membrane-based processes (Giménez-Andrés et al. 2018). They are membrane binding and often membrane remodeling modules, with hydrophobic and polar residues segregated on two opposing sides of the α-helix. Generally, the hydrophobic side is able to insert into a phospholipid monolayer, with its central axis positioned at the level of the lipid glycerol group, though varying insertion depth and angles have been observed (Ford et al. 2002; Kweon et al. 2006; Campelo et al. 2008; Yoon et al. 2010). Often, this membrane binding peptide is unstructured in solution. Membrane insertion and folding is a multi-step process (Drin and Antonny 2010). Insertion of the AH into one monolayer of a lipid membrane ultimately results in an increased surface through a wedging effect, which leads to non-zero spontaneous curvature (Zimmerberg and Kozlov 2006; Kozlov et al. 2014). In the case of CME, molecules with AH bind to the plasma membrane from the cytoplasmic side and contribute to vesicle maturation (Ford et al. 2002). For various proteins containing AH, it was not only detected that they can mold membranes but also that they are able to sense membrane curvature through lipid packing defects (Antonny 2011). The analysis of membrane curvature sensing made use of different model membrane systems. As such, curvature sensing abilities of peripheral membrane proteins were studied in detail using, for example, solid supported wavy membranes and lipid nano-tubes that are pulled out of GUVs (Roux et al. 2010; Hsieh et al. 2012).

While for some time, it was accepted by the field that peripheral membrane proteins can act on membrane morphology by either of these two mechanisms, scaffolding or wedging, more recently molecular crowding was identified as a driving force for membrane curvature induction (Stachowiak et al. 2012). The model is based on the observation that a high density of molecules, coupled to a membrane but not necessarily inserted, leads to positive membrane curvature (Stachowiak et al. 2013). In the crowding model, the energy to mold lipid membranes is generated by collisions between membrane-bound proteins. In line with this model, intrinsically disordered protein domains that are found in many proteins involved in membrane trafficking events, enhance curvature sensing and induction (Zeno et al. 2018).

It should be noted that the abovementioned molecular mechanisms for membrane molding are not mutually exclusive. N-BAR domains, for example, display an AH within on their membrane binding surface. Thus, they have the possibility to sense curvature by two different mechanisms and induce curvature by scaffolding and wedging. Consequently, N-BAR domains are popular proteins to study the biochemical and physical details of protein–membrane interaction. Similarly, it seems likely that molecular crowding works in addition to scaffolding or wedging. The below discussed CME protein epsin1, for example, was shown to use AH insertion for curvature induction but also displays a long unstructured part that could very well increase curvature by crowding (Steinem and Meinecke 2020).

### Epsin1

Epsin1 belongs to the conserved epsin family proteins that are found in many membrane trafficking pathways (Legendre-Guillemin et al. 2004). Epsin1 is involved in CME where it serves as an adaptor that bridges membrane binding and protein recruitment. Epsin1 has a modular domain structure with a tightly folded n-terminal membrane binding module (epsin n-terminal homology (ENTH) domain) and a long, mainly disordered C-terminus that comprises several protein–protein interaction motifs (Kalthoff et al. 2002; Zeno et al. 2018). Through these short peptide motifs, epsin1 is able to bind to the α-ear of the adaptor complex AP2, to intersectin, Eps15 and to clathrin heavy chains, all proteins involved in CME (Chen et al. 1998; Rosenthal et al. 1999; Overstreet et al. 2003). Additionally, epsin1 comes with ubiquitin binding motifs, most probably to recruit ubiquitinated cargo molecules destined for degradation, into clathrin-coated vesicles (Hawryluk et al. 2006; Kazazic et al. 2009).

Membrane binding and remodeling carried out by the ENTH domain is generally better understood than the exact physiological role epsins play in specific membrane trafficking pathways. Several high-resolution structures for the ENTH domain are available (Itoh et al. 2001; Ford et al. 2002). At its very N-terminus of the α-helical, globular domain an amphipathic helix is found. In solution, this part of the primary structure is disordered. The ENTH domain binds to phosphatidylinositide-containing with a preference for phosphatidylinositol-4,5-bisphosphate (PIP_2_). Binding to PIP_2_ leads to folding of the N-terminus into an AH referred to as α0. Basic residues on the polar side of the AH stabilize PIP2 binding and position the hydrophobic side that consequently inserts into the inner leaflet of the plasma membrane. Membrane binding of ENTH was shown to lead to membrane deformation (Ford et al. 2002). Additionally, using nano-tubes pulled out of GUVs by optical tweezers, a curvature sensing activity of the ENTH domain was shown (Capraro et al. 2010). Due to the protein binding-dependent membrane deformation and the amphipathic nature of α0, it was postulated that shallow insertion of α0 at one side of the bilayer leads to membrane curvature through wedging. EPR studies showing that α0 penetrated into the hydrophobic region of the lipid bilayer later confirmed this model (Kweon et al. 2006; Yoon et al. 2010).

Although, several theoretical, structural, and biochemical studies have underlined that AH insertion drives membrane remodeling by ENTH domains, the model is not without contradiction. Calculations based on a numerical solution of an analytical continuum mechanics model presented realistic conditions under which α0 of ENTH domains can drive membrane curvature (Campelo et al. 2008). Other studies found that unlikely, high non-physiological concentrations of the AH on the membrane would have to be required for α0 shallow insertion to be responsible for membrane remodeling (Stachowiak et al. 2013). Results from studies using the AH from N-BAR domains without the BAR domain did not generate significant membrane curvature (Chen et al. 2016). Recently, macromolecular crowding was suggested to be the main driving force for membrane deformation by epsin1. An ENTH domain lacking α0 was shown to induce membrane curvature at a protein coverage of a membrane above 20% (Stachowiak et al. 2012). The energy to mold membranes was supposed to be generated by collisions between membrane-bound protein.

Results from our group showed that ENTH domain membrane binding leads to decreased lateral membrane tension (Gleisner et al. 2014, 2016). This, in turn, lowers the energy barrier to induce membrane curvature (Steinem and Meinecke 2020). The mechanism of decreasing lateral membrane tension and generation of curvature depends on AH insertion and the lipid-specific oligomerization of the ENTH domain (Kroppen et al. 2020). It therefore seems reasonable to assume that ENTH-dependent membrane remodeling relies on different molecular mechanisms that combine cooperativity of protein–lipid and protein–protein interactions.

## Mitochondrial inner membrane morphology

Mitochondria execute multiple physiological functions within the cell among which are amino acids and iron clusters synthesis, lipids biogenesis and apoptosis regulation. Most importantly, mitochondria perform oxidative phosphorylation to produce the vast majority of cellular ATP. These functions, as well as the needed dynamic plasticity of mitochondria, while following the cellular needs, strongly influence mitochondrial ultrastructure and overall morphology (Mannella 2008; Zick et al. 2009; Friedman and Nunnari 2014; Barbot and Meinecke 2016). Mitochondria comprise two membranes and four sub-organellar compartments. The mitochondrial outer membrane (MOM) separates the organelle from the rest of the cytosol and is relatively even. The mitochondrial inner membrane (MIM) encloses the innermost aqueous compartment called mitochondrial matrix. As the MIM is highly folded, it has a several times larger surface area in comparison to the MOM. Together, MOM and MIM enclose another aqueous compartment called intermembrane space (IMS). The MIM can be further divided into two topologically and, importantly, functionally distinct domains of specific protein content (Mannella et al. 1994; Williams 2000; Wurm and Jakobs 2006; Strauss et al. 2008; Rabl et al. 2009; Stoldt et al. 2012). The inner boundary membrane (IBM), which runs in close proximity to the MOM and the cristae membranes (CM), which protrude toward the mitochondrial matrix. The IBM and the CM are separated by narrow, slot-like membranous structures with an inner diameter of about 15–35 nm, known as the cristae junctions (CJs) (Mannella 2006, 2008). Several remarkable morphological features of the mitochondrial inner membrane were described. These include an almost 90° angle of membrane curvature observed at the cristae junctions, long tubular/lamellar cristae stalks, and the highly bent cristae tips.

The complex organization of the MIM and, at the same time, the very dynamic nature of this organelle, both of which intimately connected to physiological functions, suggest the existence of multiple membrane curvature maintenance, as well as regulation mechanisms. Due to the apparent necessity to regulate MIM morphology to adopt to varying physiological demands, the MIM ultrastructure gained much attention over the last decade. As a result, several players were identified to be involved in the MIM morphology maintenance and remodeling.

### Players in MIM remodeling

#### *F*_*1*_*F*_*O*_* ATP synthase—cristae tips*

For a long time, it was thought that mitochondrial cristae are mere membrane folds devoted to increase the capacity of ATP production by providing the surface for large amounts of oxidative phosphorylation (OXPHOS) complexes. The understanding that the inner mitochondrial membrane is not just randomly folded but is structurally highly regulated started with the investigation of the OXPHOS complex V, also known as F_1_F_O_ ATP synthase, and its organization within the MIM. Early electron microscopy studies of negatively stained cristae membranes in bovine cardiac muscle cells revealed very characteristic 9 nm lollipop-like globular protrusions of ATP synthase localized to the cristae membrane and facing the mitochondrial matrix (Fernandez-Moran 1962). For some time, it remained a matter of debates whether these structures are corresponding to the native orientation in the membrane or whether they are artifacts introduced by the application of negative stain. This was out of the question when these structures were re-investigated using fast-freeze/deep-etch electron microscopy followed by rotatory shadowing using whole cells, as well as isolated mitochondria of *Paramecium* (Allen et al. 1989). This method of sample preparation allows to acquire detailed three-dimensional images of macromolecular moieties in almost native state avoiding common artifacts of conventional electron microscopy. Similar topological arrangement of ATP synthase molecules, as well as their highly ordered distribution within the cristae membranes were observed (Allen et al. 1989). The characteristic lollipop-shaped ATP synthase molecules were organized in double rows along the cristae stalk. It was then, when the first models of tubular cristae formation via ATP synthase dimerization were suggested (Allen et al. 1989; Paumard et al. 2002). Meanwhile, it is known that the enzymatically active mitochondrial ATP synthase, in contrast to its bacterial counterpart, forms dimers (Arnold et al. 1998). It was shown using native BN-PAGE followed by high-resolution second dimension SDS-PAGE that dimerization of ATP synthase relies on auxiliary transmembrane subunits (Su) of the F_O_-domain Su e, Su g, and Su k (Arnold et al. 1998). Later it was also shown that dimerization and further oligomerization of ATP synthase dimers into higher order oligomers through Su e and Su g is the crucial point in the formation curved cristae tips/rims (Paumard et al. 2002; Strauss et al. 2008). Null mutant yeast cells missing either Su e or Su g possess only monomers of ATP synthase. Although, the monomeric ATP synthases still localizes to the MIM, changed mitochondrial ultrastructure was found using electron microscopy. Unregulated folding of the MIM resulted in formation of onion-like structures instead of tubular cristae (Paumard et al. 2002). According to current views, ATP synthase dimers resemble truncated cones, which serve as a rigid arc-shaped scaffold and promote strong local membrane curvature, hence inward protrusion of the MIM (Paumard et al. 2002; Davies et al. 2012; Kühlbrandt 2019). Moreover, this process is further supported by the association of additional ATP synthase dimers into long ribbon-like super-complexes (Strauss et al. 2008). Cross-linking studies showed that ATP synthase dimers are formed via membrane-embedded interface between subunit e and g mediated through GXXXG motifs (Habersetzer et al. 2013). Su e or su g knock out in yeast, as well us amino acid substitutions in the GXXXG motif, distorts the normal process of MIM biogenesis and leads to random folding which results in multi-lamellar, onion-like structures instead of tubular cristae (Arselin et al. 2003; Bustos and Velours 2005). Single-particle electron microscopy, as well as cryo-electron tomography measurements in whole mitochondria suggested a 70° angle between long axes of ATPase single molecules in the dimers (Davies et al. 2011). The fact that membrane patches produced after disruption of mitochondria still maintain the curved morphology rather confirms the idea that the dimer rows shape the cristae rims rather than the other way around.

#### Mgm1/OPA1—cristae membranes

The fungal mitochondrial genome maintenance protein (Mgm1), in animals known as optical atrophy 1 protein (OPA1), is a large GTPase of the dynamin superfamily that is localized to the MIM. Members of this protein family are involved in various membrane trafficking and membrane remodeling processes, and Mgm1/OPA1 is one of the central elements in remodeling of the MIM (Ferguson and De Camilli 2012). More precisely, it is involved in the MIM fusion, as well as in regulation of the cristae membrane morphology (Pellegrini and Scorrano 2007; Hoppins and Nunnari 2009). According to results from immunofluorescence and confocal microscopy 3D-reconstitution of mitochondria, downregulation of Mgm1/OPA1 leads to fragmentation of the mitochondrial network, disorganization of the inner mitochondrial membrane and loss of mitochondrial genomic DNA (Jones and Fangman 1992; Olichon et al. 2003). More detailed investigation of the MIM ultrastructure of such cells by transmission EM revealed unstructured vesicular cristae stalks with abnormally large distances between the membranes (Olichon et al. 2003; Frezza et al. 2006).

Mature Mgm1 is anchored into the lipid bilayer of the MIM via a single transmembrane domain and exposes a large soluble part into the IMS. The IMS domain of Mgm1/OPA1 comprises four domains: GTPase (G-domain), bundle signaling element (BSE), stalk, and membrane-interacting paddle (Faelber et al. 2019). Further, proteolytic processing by an IMS-localized rhomboid-like protease in the region between the transmembrane domain and the G-domain renders two Mgm1-isoforms: soluble short Mgm1 (S-Mgm1), as well as membrane-bound long Mgm1 (L-Mgm1) (Amutha et al. 2004; Ishihara et al. 2006). A combination of both, S- and L-Mgm1 isoforms is required for mitochondrial fusion, while only S-Mgm1 is required for cristae membrane remodeling (Ishihara et al. 2006; Ban et al. 2010).

Liposome co-sedimentation assays with recombinantly purified short isoform of OPA1 revealed a strong binding preference to the negatively charged liposomes containing, for example, phosphatidylserine (PS) or cardiolipin (CL) (Ban et al. 2010). Membrane binding promoted the rather low basal GTPase activity of OPA1 up to 100 folds, as well as assembly of OPA1 into higher order oligomers, as detected by homo-bifunctional cross-linking experiments (Ban et al. 2010; Zhang et al. 2020). Fluorescence microscopy, negative stain transmission EM, as well as electron cryo-microscopy showed that these higher order oligomers assemble into a regular pattern and induce protrusion of lipid tubules from the cardiolipin-containing liposomes (Frezza et al. 2006; Ban et al. 2010; Zhang et al. 2020).

Recent functional and structural studies performed on the fungal S-Mgm1 revealed similar behavior of the protein, as well as shed the light onto the molecular principles of Mgm1 oligomerization and assembly into regular patterns on the membrane (Faelber et al. 2019). Using X-ray crystallography, it was observed that Mgm1 forms V-shaped dimers assembled via conserved hydrophobic interface of the stalk domains. Mgm1’s ability to dimerize was shown to be important for its membrane binding ability, assembly into a regular pattern on the membrane surface, as well as for membrane deformation. When expressed in *Saccharomyces cerevisiae* cells lacking endogenous Mgm1, dimerization mutants were not able to rescue Mgm1 protein function, which was assessed by growing on non-fermentable medium, fluorescence microscopy of the mitochondria of corresponding strains, as well as blotting against proteins encoded in mitochondrial DNA (Faelber et al. 2019).

In the same study, the structure of Mgm1 bound to positively, as well as negatively curved membranes was solved using cryo-ET sub-tomogram averaging (Faelber et al. 2019). It provided more details to the earlier observations of Mgm1 decorating membrane tubules in a regular pattern (Ban et al. 2010). According to the cryo-ET results, Mgm1 can assemble on the inside of the liposomal membrane (negative membrane curvature, topologically and geometrically resembling the inside of the narrow tubular parts of mitochondrial cristae junctions) to form helical structures, where the paddle domain interacts with the membrane, stalks are positioned in the middle while BSE and G-domains are exposed to the lumen of the membrane tubule. The backbone of the helical filament is formed by the stalk domains, and the contacts between adjacent helix turns is mediated through the G-domains (Faelber et al. 2019). Cryo-EM structures of S-Mgm1 assemblies at positively curved membranes revealed similar membrane organization of S-Mgm1 dimers: Paddle-Stalk-BSE-G-domain, while the interaction between dimers of adjacent filament turns was mediated via paddle domains and G-domains staying far apart from each other. Interestingly, results of live fluorescence microscopy of GUVs manipulated with optical tweezer showed that Mgm1 was concentrating at the connection point of the GUV surface and the tubular invagination while slowly growing along the tubule during the course of incubation (Faelber et al. 2019).

Based on the sequence and structure similarities between the G-domains and BSE domains of Mgm1 and dynamin, similar mechanisms of GTPase activity stimulation were proposed. In this model Mgm1, like dynamin, undergoes a power stroke upon GTP hydrolysis to remodel membranes. This power stroke would result in different remodeling processes depending on the assembly geometry of Mgm1. A power stroke in left-handed helix on negatively curved membranes would result in constriction of its diameter—as, for instance, in cristae membranes upon OPA1 overexpression, whereas a power stroke of right-handed helix would lead to the tubule expansion (Faelber et al. 2019). Taking these models into account, one could assume that on the inside of mitochondrial CJs, Mgm1 can assemble into a left-handed helical filament and upon GTP hydrolysis constrict the diameter of the CM or the CJs. This would, in turn, seclude the mitochondrial pool of cytochrome c and / or maintaining the respiratory active conformation of the cristae membrane. When assembled into a right-handed filament, Mgm1 could compensate for the tubulation force of ATP synthase dimers at the cristae tips and MICOS proteins at the cristae junctions preventing the cristae membranes from collapsing onto each other (Faelber et al. 2019).

Parallel investigations of the cryo-EM structure of liposomes coated with human S-OPA1, as well as X-ray crystallography of the yeast S-Mgm1 support the findings made about fungal Mgm1 protein regarding the importance of its oligomerization, GTPase function activation, domain structure, and its membrane curvature induction activity (Zhang et al. 2020). Sequence analysis of the human S-OPA1 as well as yeast S-Mgm1 suggest that their C-terminal domains (corresponding to the paddle domain of Chaetomium Mgm1) exert membrane binding and deformation via an AH. Mutagenesis experiments, in which AH formation was hindered by amino acid substitutions showed that AH mutants had decreased membrane binding affinity, GTPase activity, as well as tubulation activity.

#### Lipids (PE and CL)

The major structural lipids of eukaryotic cell membranes are glycerophospholipids with various headgroups: phosphatidylcholine (PC), phosphatidylethanolamine (PE), phosphatidylserine PS, phosphatidylinositol (PI), and phosphatidic acid (PA) (van Meer et al. 2008). They all have different geometry based on the size and shape of their headgroups and fatty acid tail components. PC, PS, and PI have overall cylindrical shape and promote formation of flat planar lipid bilayers. The hydrophilic head of PE is smaller and together with its poly-acyl tail adopts a conical shape. When enriched in membranes, such shape would promote curvature stress, which is utilized in such cellular processes as membrane fission and fusion and membrane budding (Cullis and de Kruijff 1979; Verkleij et al. 1984; Epand et al. 1996; Siegel and Epand 1997; Brink-van der Laan et al. 2004).

Lipids are not randomly distributed between cellular membranes. There is a large degree of lipid asymmetry between different organelles (van Meer 2005), which is even true for membranes and/or bilayer leaflets within the same organelle. A mitochondrion is a good example to illustrate that, since it is enclosed by two membranes. The MOM is relatively flat and consists of 44–59% PC, 20–35% PE, 5–20% PI with rest amount taken over by PS, PA, and lysophospholipids. The MIM consists of 38–45% PC, 32–39% PE, 2–7% PI, 14–23% CL (van Meer et al. 2008). Cardiolipin (CL) is mitochondria-specific lipid that is essentially consisting of two PA molecules. CL is large, conical non-bilayer lipid similarly to PE (Cullis and de Kruijff 1979; van Meer 2005; Basu Ball et al. 2018). In this way, the MIM can consist up to 62% (over 32% of MOM) of non-bilayer lipids, which is in line with its convoluted, highly curved morphology.

### MICOS—cristae junctions

**Fig. 2:**
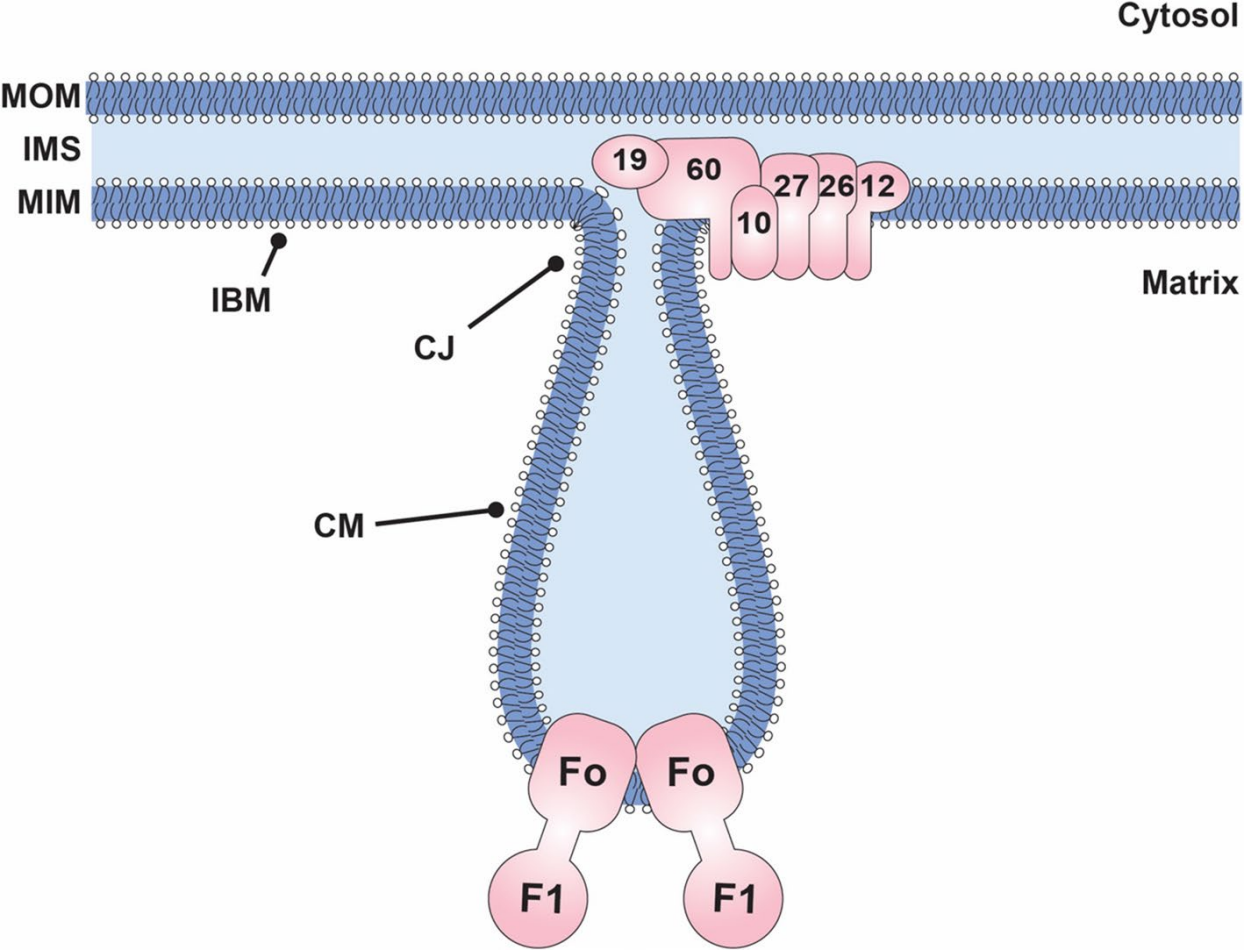
Protein-dependent mitochondrial inner membrane morphology. The schematic illustration of a part of a mitochondrion shows two major complexes (MICOS and F_1_F_O_ ATP synthase) of the inner membrane (IM) that have been identified to directly affect cristae membrane (CM) formation. While the mitochondrial outer membrane (MOM) is relatively smooth, the MIM shows two morphological distinct subdomains: inner boundary membrane (IBM) and CM that are connected by cristae junction (CJ). Intermembrane space (IMS)

The mitochondrial contact sites and cristae organizing system (MICOS) resides at mitochondrial cristae junctions. This multi-subunit protein complex is found in all eukaryotic organisms containing mitochondria (Muñoz-Gómez et al. 2015; Huynen et al. 2016). MICOS consists of six membrane-associated protein subunits named Mic60, Mic27, Mic26, Mic19, Mic12, and Mic10 (Pfanner et al. 2014). In mammals, there is an additional subunit, paralogous to Mic19, called Mic25. All MICOS subunits contain at least one transmembrane domain apart from the Mic19/Mic25 proteins, which interact with the MIM peripherally. Deletion of single MICOS components leads to drastic changes in MIM morphology characterized by the loss of CJs, pinching off the cristae membranes from the IBM and as a result, their accumulation within the mitochondrial matrix in the shape of enclosed membrane sacks (John et al. 2005; Hoppins et al. 2011; Harner et al. 2011; Malsburg et al. 2011). Derived from the severity of these protein deletion effects, Mic10 and Mic60 were defined as the two core subunits of MICOS. Both proteins are evolutionary conserved within the kingdom of eukaryotes with Mic60 being the oldest subunit of the complex (Muñoz-Gómez et al. 2015). Altogether, MICOS is thought to maintain the cristae junctions balancing membrane curvature at the cristae rims induced by the ATP synthase oligomers (Barbot and Meinecke 2016; Rampelt and van der Laan 2017) (Fig. 2).

#### Mic10

Mic10 is the smallest, but the most abundant component of the MICOS complex. Studies on Mic10’s involvement in the maintenance of the MIM morphology began from the investigation of mitochondrial ultrastructure of the knock-out cells, as well as from overexpression experiments (Hoppins et al. 2011; Harner et al. 2011; Malsburg et al. 2011). Based on negative stain TEM studies of the mitochondrial ultrastructure, it was found that Mic10 deletion mutants lose the CJ structures and accumulate the CM if the form of piled up membrane stacks, while cells with elevated Mic10 expression levels exhibit deformed CJ structures and elongated cristae membranes. Already, these observations pointed toward the particular importance of Mic10 for the maintenance of proper MIM morphology.

According to the secondary structure prediction, the 10 kDa protein consists of two transmembrane helices. The first helix exhibits typical parameters of standard transmembrane domain, while the second helix appears to be several amino acid residues longer. Both helices contain conserved sequence motifs comprising consecutive glycine residues (Alkhaja et al. 2012). PEGylation of relevant Mic10 cysteine mutants, as well as protease K treatment of yeast mitoplast expressing C- and N-terminally tagged Mic10 variant showed that Mic10 adopts a hairpin-like topology within the MIM with N-/C-termini exposed to the IMS (Barbot et al. 2015; Bohnert et al. 2015). A membrane remodeling activity of Mic10 was shown by direct visualization of the Mic10-containing vesicles (Barbot et al. 2015). Negative staining TEM of the Mic10-containg liposomes revealed that Mic10 converted spherical liposomes into elongated tubules. These observations were supported by dynamic light scattering (DLS) analysis of liposomes in the absence and presence of Mic10. Additionally, Mic10-containing GUVs, analyzed by fluorescence confocal microscopy, exhibited multiple cristae-like invaginations of the membrane.

**Fig. 3:**
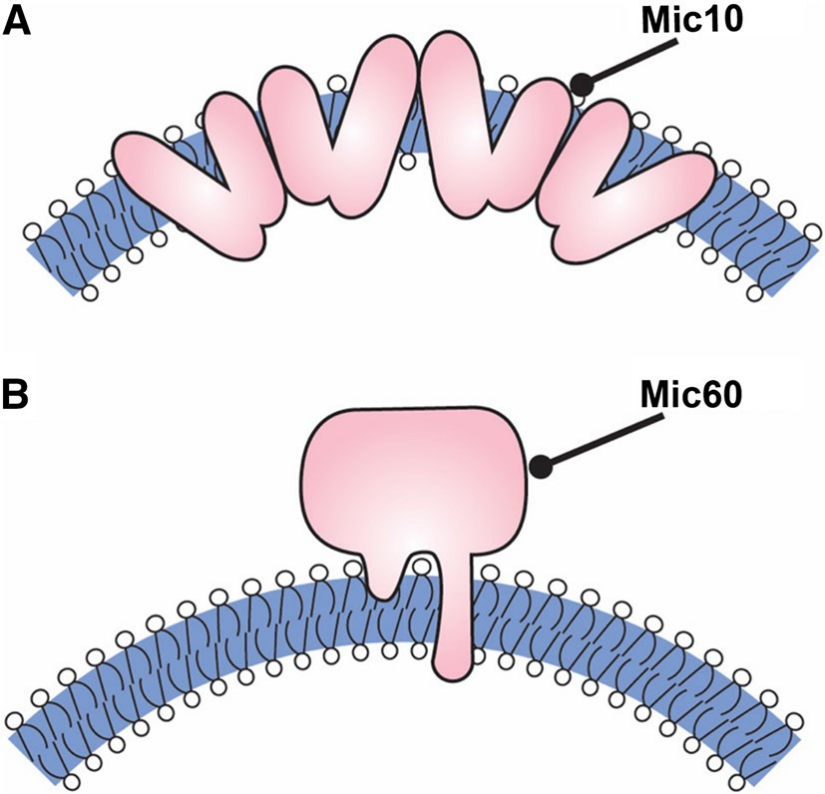
MICOS core components Mic10 and Mic60 induce membrane curvature. Model of Mic10 (**a**) and Mic60 (**b**) -dependent membrane deformation. **a** Mic10 oligomerizes and might adopt a wedge-like structure within the inner membrane. **b** Mic60 is anchored in the MIM by a single transmembrane domain. It most likely induces curvature by amphipathic helix insertion

As glycine-rich protein motives are crucial for helix–helix interaction and transmembrane segments packing within lipid bilayers (Russ and Engelman 2000), it was suggested that Mic10 might undergo homo-oligomerization. Using helical wheel projections, it was shown that GxxxG motifs are positioned on the opposite sides of the alpha-helices, thus enabling stable association of several protein molecules next to each other. This suggestion was further confirmed by the results of BN-PAGE, as well as FRET using the recombinant Mic10 protein reconstituted into liposomes (Barbot et al. 2015; Bohnert et al. 2015). Mutagenesis of the relevant amino acid residues, which disrupt GxxxG motifs of the Mic10 protein inhibited formation of the high-order oligomers in vitro. Such behavior of the mutant protein was also observed in organello experiments. Negative stain TEM of Δmic10 mitochondria expressing Mic10 oligomerization mutants exhibited a typical Δmic10 phenotype accompanied by CJs loss and piling up of enclosed cristae membranes in the matrix (Barbot et al. 2015; Bohnert et al. 2015). Notably, oligomerization mutants failed to induce tubule formation in both types of the model membranes (Barbot et al. 2015). Hence, Mic10 oligomerization is essential for its membrane remodeling activity and is a prerequisite for formation of the cristae junctions. Considering its topology and transmembrane domain properties, it was suggested that Mic10 occupies a larger surface on the outer leaflet of the inner membrane bilayer, causing elastic stress and promoting membrane deformation (Barbot et al. 2015). The GxxxG domains promote oligomerization of Mic10 on the membrane which covers energy costs of membrane bending. In this way, Mic10 is employing wedging mechanisms for membrane curvature induction, as well as oligomerization to enhance and stabilize membrane curvature (Fig. 3).

#### Mic60

Mic60 is the largest and evolutionary, the oldest component of the MICOS complex (Muñoz-Gómez et al. 2015, 2017; Huynen et al. 2016). In fact, it is the only MICOS subunit with homologs in Prokaryotes (Muñoz-Gómez et al. 2015). Deletion of Mic60, similarly to deletion of Mic10, leads to disruption of the CJ structures and disconnection of the cristae from the inner boundary membrane. Mic60’s overexpression, in contrast to Mic10, leads to an increase of CJ number, as well as to cristae branching, which rarely occurs in normal mitochondria (John et al. 2005; Rabl et al. 2009; Hoppins et al. 2011; Harner et al. 2011; Malsburg et al. 2011).

Secondary structure prediction suggests that the mature Mic60 protein consists of single N-terminal transmembrane domain, large central coiled coil domain and C-terminal signature mitofilin domain. Based on the results of proteinase K treatment of yeast mitoplasts, the N-terminal transmembrane domain of Mic60 is anchored into the MIM, while the coiled coil and the mitofilin domains are exposed to the mitochondrial IMS (Rabl et al. 2009). Co-expression of full-length Mic60 variants with different C-terminal fusion tags followed by tandem affinity purification, as well as results of the equilibrium analytical ultracentrifugation and BN-PAGE of recombinant N-terminally truncated variant suggest that Mic60 exhibits a tendency to homotypical interactions predominantly existing in a dimeric form with only small fraction assembled into higher order oligomers (Rabl et al. 2009; Hessenberger et al. 2017).

In vitro reconstitution studies using recombinantly purified full-length Mic60 revealed its membrane bending activity (Tarasenko et al. 2017). This activity was observed in artificial model membranes of different dimensions. Incorporated into LUVs, Mic60 was able to convert spherical 200-nm-sized vesicles into long tubules which were visualized by negative stain TEM. In larger, up to several micrometer-sized GUVs visualized by light confocal fluorescence microscopy Mic60 induced formation of internal vesicles, as well as interconnected membrane sheets. Moreover, Mic60 was able to form cristae-like structures de novo in living cells when targeted to the inner membrane of Gram-negative bacteria (Tarasenko et al. 2017).

According to liposome co-sedimentation, as well as liposome flotation assays, Mic60’s ability to bind and remodel lipid membranes seems to be independent of its transmembrane domain (Tarasenko et al. 2017; Hessenberger et al. 2017). LUVs incubated with N-terminally truncated variant of Mic60 were converted into long tubules as revealed by negative stain TEM. This observation in line with an in vivo experiment where IMS part of Mic60 was targeted to the periplasm of Gram-negative bacteria and induced formation of long tubular cristae-like protrusions into the bacterial cytoplasm (Tarasenko et al. 2017).

Using secondary structure prediction tools, it was identified that the Mic60 protein contains two predicted AH, designated LBS1 and LBS2, in the region between its coiled coil and mitofilin domains (Hessenberger et al. 2017). Helical wheel projections showed conserved amphipathic properties of LBS1, which is a signature feature of membrane-inserting AH, as discussed above. A Mic60 variant lacking the LBS1 sequence was not able to bind and tubulate liposomes (Hessenberger et al. 2017). Two conserved polar residues Arg572 and Phe573 appeared to be crucial for these functions of Mic60, where arginine is potential binding partner for negatively charged lipid heads, while Phenylalanine might be important for the insertion into the hydrophobic core of the lipid bilayer. Substitution of these residues by Aspartate led to the considerable reduction of lipid binding, as well as weakened liposome binding. Simultaneous substitution of both residues to Aspartate completely abolished membrane binding and tubulation. These residues appear to play a crucial role for MICOS stability. When relevant mutants were expressed in yeast and the whole MICOS was isolated, it was observed that the subunits were strongly reduced (Hessenberger et al. 2017).

Taking all together, Mic60 uses a different mechanism to bind and remodel the mitochondrial inner membrane in comparison to Mic10. In fact, this protein, while residing permanently in the MIM, used a similar membrane molding mechanism as proteins found in membrane trafficking events like CME. AH are known to sense and induce positive membrane curvature. They insert into the lipid bilayer via intercalation into one membrane leaflet competing for the space with the lipid head groups and eventually such asymmetrical insertion leads to curvature of the membrane. Usually, such curved membrane is additionally stabilized by rigid protein scaffold that supports the form of the underlying membrane e.g., BAR proteins or endophilins. Long, rod-shaped coiled coil domain of Mic60 together with amphipathic nature of its LBS1might employ similar mechanisms to shape the mitochondrial inner membrane at the CJ sites.

As expected for a dynamic process like CME, where membrane remodeling takes place on a timescale of a few seconds, membrane curvature inducing molecules are only transiently attached and are recruited, and most probably released in a spatiotemporal regulated fashion. For a process of this importance, it is also not surprising that many membrane shaping proteins with partially overlapping function are found. In addition to scaffolding and shallow insertion of proteins on membranes, molecular crowding is likely contributing to the morphological maturation of clathrin-coated vesicles. In the future, it will be important to study cooperativity of protein–protein and protein–membrane interactions in CME. As for studying the physiological role of specific lipids, these experiments are much more complex and rely on continuous exciting progresses in model membrane systems and structural investigation of biological samples.

The morphology of organellar membranes like mitochondria or the endoplasmic reticulum is, although able to dynamically respond to different cellular needs, steadier than what is observed in membrane trafficking events. Consequently, the few organelle shaping proteins that have been identified so far are, in most cases, integral membrane proteins, that reside constantly within the membrane. Due to their physical–chemical properties, in vitro investigations of these hydrophobic molecules are more demanding than for soluble proteins. A common mechanism by which membrane proteins induce curvature seems to be sterically driven. Here, the molecules cover different surface areas on each side of the membrane and, as such, adopt a wedge-like topology. Interestingly, integral membrane proteins that carry AH at their soluble domains, with which they mold membranes, have also been identified. Shallow insertion of protein moieties seems to be a general theme in the remodeling of biological membranes. Prospective studies will surely identify more membrane bending proteins and molecular mechanisms. It will be exciting to see more similarities and differences between soluble and integral membrane proteins and their effect on membrane morphology uncovered.

## References

[CR1] Alkhaja AK, Jans DC, Nikolov M (2012). MINOS1 is a conserved component of mitofilin complexes and required for mitochondrial function and cristae organization. Mol Biol Cell.

[CR2] Allen RD, Schroeder CC, Fok AK (1989). An investigation of mitochondrial inner membranes by rapid-freeze deep-etch techniques. J Cell Biol.

[CR3] Amutha B, Gordon DM, Gu Y, Pain D (2004). A novel role of Mgm1p, a dynamin-related GTPase, in ATP synthase assembly and cristae formation/maintenance. Biochem J.

[CR4] Antonny B (2011). Mechanisms of membrane curvature sensing. Annu Rev Biochem.

[CR5] Arnold I, Pfeiffer K, Neupert W (1998). Yeast mitochondrial F1F0-ATP synthase exists as a dimer: identification of three dimer-specific subunits. EMBO J.

[CR6] Arselin G, Giraud M-F, Dautant A (2003). The GxxxG motif of the transmembrane domain of subunit e is involved in the dimerization/oligomerization of the yeast ATP synthase complex in the mitochondrial membrane. Eur J Biochem.

[CR7] Ban T, Heymann JAW, Song Z (2010). OPA1 disease alleles causing dominant optic atrophy have defects in cardiolipin-stimulated GTP hydrolysis and membrane tubulation. Hum Mol Genet.

[CR8] Barbot M, Meinecke M (2016). Reconstitutions of mitochondrial inner membrane remodeling. J Struct Biol.

[CR9] Barbot M, Jans DC, Schulz C (2015). Mic10 oligomerizes to bend mitochondrial inner membranes at cristae junctions. Cell Metab.

[CR10] Basu Ball W, Neff JK, Gohil VM (2018). The role of nonbilayer phospholipids in mitochondrial structure and function. FEBS Lett.

[CR11] Baumgart T, Capraro BR, Zhu C, Das SL (2011). Thermodynamics and mechanics of membrane curvature generation and sensing by proteins and lipids. Annu Rev Phys Chem.

[CR12] Bohnert M, Zerbes RM, Davies KM (2015). Central role of Mic10 in the mitochondrial contact site and cristae organizing system. Cell Metab.

[CR13] Bustos DM, Velours J (2005). The modification of the conserved GXXXG motif of the membrane-spanning segment of subunit g destabilizes the supramolecular species of yeast ATP synthase. J Biol Chem.

[CR14] Campelo F, McMahon HT, Kozlov MM (2008). The hydrophobic insertion mechanism of membrane curvature generation by proteins. Biophys J.

[CR15] Capraro BR, Yoon Y, Cho W, Baumgart T (2010). Curvature sensing by the epsin N-terminal homology domain measured on cylindrical lipid membrane tethers. J Am Chem Soc.

[CR16] Chen H, Fre S, Slepnev VI (1998). Epsin is an EH-domain-binding protein implicated in clathrin-mediated endocytosis. Nature.

[CR17] Chen Z, Zhu C, Kuo CJ (2016). The N-terminal amphipathic helix of endophilin does not contribute to its molecular curvature generation capacity. J Am Chem Soc.

[CR18] Cullis PR, de Kruijff B (1979). Lipid polymorphism and the functional roles of lipids in biological membranes. Biochim Biophys Acta.

[CR19] Daumke O, Roux A, Haucke V (2014). BAR domain scaffolds in dynamin-mediated membrane fission. Cell.

[CR20] Davies KM, Strauss M, Daum B (2011). Macromolecular organization of ATP synthase and complex I in whole mitochondria. Proc Natl Acad Sci USA.

[CR21] Davies KM, Anselmi C, Wittig I (2012). Structure of the yeast F1Fo-ATP synthase dimer and its role in shaping the mitochondrial cristae. Proc Natl Acad Sci USA.

[CR22] Doherty GJ, McMahon HT (2009). Mechanisms of endocytosis. Annu Rev Biochem.

[CR23] Drin G, Antonny B (2010). Amphipathic helices and membrane curvature. FEBS Lett.

[CR24] Epand RM, Fuller N, Rand RP (1996). Role of the position of unsaturation on the phase behavior and intrinsic curvature of phosphatidylethanolamines. Biophys J.

[CR25] Faelber K, Dietrich L, Noel JK (2019). Structure and assembly of the mitochondrial membrane remodelling GTPase Mgm1. Nature.

[CR26] Farsad K, Ringstad N, Takei K (2001). Generation of high curvature membranes mediated by direct endophilin bilayer interactions. J Cell Biol.

[CR27] Ferguson SM, De Camilli P (2012). Dynamin, a membrane-remodelling GTPase. Nat Rev Mol Cell Biol.

[CR28] Fernandez-Moran H (1962). Cell-membrane ultrastructure. Low-temperature electron microsopy and x-ray diffraction studies of lipoprotein components in lamellar systems. Circulation.

[CR29] Ford MG, Mills IG, Peter BJ (2002). Curvature of clathrin-coated pits driven by epsin. Nature.

[CR30] Frezza C, Cipolat S, Martins de Brito O (2006). OPA1 controls apoptotic cristae remodeling independently from mitochondrial fusion. Cell.

[CR31] Friedman JR, Nunnari J (2014). Mitochondrial form and function. Nature.

[CR32] Frost A, Perera R, Roux A (2008). Structural basis of membrane invagination by F-BAR domains. Cell.

[CR33] Frost A, Unger VM, De Camilli P (2009). The BAR domain superfamily: membrane-molding macromolecules. Cell.

[CR34] Gallop JL, Jao CC, Kent HM (2006). Mechanism of endophilin N-BAR domain-mediated membrane curvature. EMBO J.

[CR35] Giménez-Andrés M, Čopič A, Antonny B (2018). The many faces of amphipathic helices. Biomolecules.

[CR36] Gleisner M, Mey I, Barbot M (2014). Driving a planar model system into the 3(rd) dimension: generation and control of curved pore-spanning membrane arrays. Soft Matter.

[CR37] Gleisner M, Kroppen B, Fricke C (2016). Epsin N-terminal homology domain (ENTH) activity as a function of membrane tension. J Biol Chem.

[CR38] Habersetzer J, Ziani W, Larrieu I (2013). ATP synthase oligomerization: from the enzyme models to the mitochondrial morphology. Int J Biochem Cell Biol.

[CR39] Harner M, Körner C, Walther D (2011). The mitochondrial contact site complex, a determinant of mitochondrial architecture. EMBO J.

[CR40] Haucke V, Kozlov MM (2018). Membrane remodeling in clathrin-mediated endocytosis. J Cell Sci.

[CR41] Hawryluk MJ, Keyel PA, Mishra SK (2006). Epsin 1 is a polyubiquitin-selective clathrin-associated sorting protein. Traffic.

[CR42] Henne WM, Kent HM, Ford MG (2007). Structure and analysis of FCHo2 F-BAR domain: a dimerizing and membrane recruitment module that effects membrane curvature. Structure.

[CR43] Henne WM, Boucrot E, Meinecke M (2010). FCHo proteins are nucleators of clathrin-mediated endocytosis. Science.

[CR44] Hessenberger M, Zerbes RM, Rampelt H (2017). Regulated membrane remodeling by Mic60 controls formation of mitochondrial crista junctions. Nat Commun.

[CR45] Hoppins S, Nunnari J (2009). The molecular mechanism of mitochondrial fusion. Biochim Biophys Acta.

[CR46] Hoppins S, Collins SR, Cassidy-Stone A (2011). A mitochondrial-focused genetic interaction map reveals a scaffold-like complex required for inner membrane organization in mitochondria. J Cell Biol.

[CR47] Hsieh W-T, Hsu C-J, Capraro BR (2012). Curvature sorting of peripheral proteins on solid-supported wavy membranes. Langmuir.

[CR48] Huynen MA, Mühlmeister M, Gotthardt K (2016). Evolution and structural organization of the mitochondrial contact site (MICOS) complex and the mitochondrial intermembrane space bridging (MIB) complex. Biochim Biophys Acta.

[CR49] Ishihara N, Fujita Y, Oka T, Mihara K (2006). Regulation of mitochondrial morphology through proteolytic cleavage of OPA1. EMBO J.

[CR50] Itoh T, Koshiba S, Kigawa T (2001). Role of the ENTH domain in phosphatidylinositol-4,5-bisphosphate binding and endocytosis. Science.

[CR51] John GB, Shang Y, Li L (2005). The mitochondrial inner membrane protein mitofilin controls cristae morphology. Mol Biol Cell.

[CR52] Jones BA, Fangman WL (1992). Mitochondrial DNA maintenance in yeast requires a protein containing a region related to the GTP-binding domain of dynamin. Genes Dev.

[CR53] Kalthoff C, Alves J, Urbanke C (2002). Unusual structural organization of the endocytic proteins AP180 and epsin 1. J Biol Chem.

[CR54] Kazazic M, Bertelsen V, Pedersen KW (2009). Epsin 1 is involved in recruitment of ubiquitinated EGF receptors into clathrin-coated pits. Traffic.

[CR55] Kirchhausen T, Owen D, Harrison SC (2014). Molecular structure, function, and dynamics of clathrin-mediated membrane traffic. Cold Spring Harb Perspect Biol.

[CR56] Kozlov MM, Campelo F, Liska N (2014). Mechanisms shaping cell membranes. Curr Opin Cell Biol.

[CR104] Kroppen B, Teske N, Yambire KF, Denkert N, Mukherjee I, Tarasenko D, Jaipuria G, Zweckstetter M, Milosevic I, Steinem C, Meinecke M (2020) Cooperativity of membrane-protein and protein-protein interactions control membrane remodeling by epsin 1 and affects clathrin-mediated endocytosis. Cell Mol Life Sci. 10.1007/s00018-020-03647-z 10.1007/s00018-020-03647-z PMC7966211 32997199

[CR57] Kühlbrandt W (2019). Structure and mechanisms of F-Type ATP synthases. Annu Rev Biochem.

[CR58] Kweon DH, Shin YK, Shin JY (2006). Membrane topology of helix 0 of the Epsin N-terminal homology domain. Mol Cells.

[CR59] Legendre-Guillemin V, Wasiak S, Hussain NK (2004). ENTH/ANTH proteins and clathrin-mediated membrane budding. J Cell Sci.

[CR60] Mannella CA (2006). Structure and dynamics of the mitochondrial inner membrane cristae. Biochim Biophys Acta.

[CR61] Mannella CA (2008). Structural diversity of mitochondria: functional implications. Ann N Y Acad Sci.

[CR62] Mannella CA, Marko M, Penczek P (1994). The internal compartmentation of rat-liver mitochondria: tomographic study using the high-voltage transmission electron microscope. Microsc Res Tech.

[CR63] McMahon HT, Gallop JL (2005). Membrane curvature and mechanisms of dynamic cell membrane remodelling. Nature.

[CR64] Meinecke M, Boucrot E, Camdere G (2013). Cooperative recruitment of dynamin and BIN/Amphiphysin/Rvs (BAR) domain-containing proteins leads to GTP-dependent membrane scission. J Biol Chem.

[CR65] Muñoz-Gómez SA, Slamovits CH, Dacks JB (2015). Ancient homology of the mitochondrial contact site and cristae organizing system points to an endosymbiotic origin of mitochondrial cristae. Curr Biol.

[CR66] Muñoz-Gómez SA, Wideman JG, Roger AJ, Slamovits CH (2017). The origin of mitochondrial cristae from alphaproteobacteria. Mol Biol Evol.

[CR67] Olichon A, Baricault L, Gas N (2003). Loss of OPA1 perturbates the mitochondrial inner membrane structure and integrity, leading to cytochrome c release and apoptosis. J Biol Chem.

[CR68] Overstreet E, Chen X, Wendland B, Fischer JA (2003). Either part of a Drosophila epsin protein, divided after the ENTH domain, functions in endocytosis of delta in the developing eye. Curr Biol.

[CR69] Paumard P, Vaillier J, Coulary B (2002). The ATP synthase is involved in generating mitochondrial cristae morphology. EMBO J.

[CR70] Pellegrini L, Scorrano L (2007). A cut short to death: Parl and Opa1 in the regulation of mitochondrial morphology and apoptosis. Cell Death Differ.

[CR71] Peter BJ, Kent HM, Mills IG (2004). BAR domains as sensors of membrane curvature: the amphiphysin BAR structure. Science.

[CR72] Pfanner N, van der Laan M, Amati P (2014). Uniform nomenclature for the mitochondrial contact site and cristae organizing system. J Cell Biol.

[CR73] Rabl R, Soubannier V, Scholz R (2009). Formation of cristae and crista junctions in mitochondria depends on antagonism between Fcj1 and Su e/g. J Cell Biol.

[CR74] Rampelt H, van der Laan M (2017). The Yin & Yang of Mitochondrial Architecture - Interplay of MICOS and F1Fo-ATP synthase in cristae formation. Microb Cell.

[CR75] Rosenthal JA, Chen H, Slepnev VI (1999). The epsins define a family of proteins that interact with components of the clathrin coat and contain a new protein module. J Biol Chem.

[CR76] Roux A, Koster G, Lenz M (2010). Membrane curvature controls dynamin polymerization. Proc Natl Acad Sci USA.

[CR77] Russ WP, Engelman DM (2000). The GxxxG motif: a framework for transmembrane helix-helix association. J Mol Biol.

[CR78] Saarikangas J, Zhao H, Pykalainen A (2009). Molecular mechanisms of membrane deformation by I-BAR domain proteins. Curr Biol.

[CR79] Saheki Y, De Camilli P (2012). Synaptic vesicle endocytosis. Cold Spring Harb Perspect Biol.

[CR80] Shimada A, Niwa H, Tsujita K (2007). Curved EFC/F-BAR-domain dimers are joined end to end into a filament for membrane invagination in endocytosis. Cell.

[CR81] Siegel DP, Epand RM (1997). The mechanism of lamellar-to-inverted hexagonal phase transitions in phosphatidylethanolamine: implications for membrane fusion mechanisms. Biophysj.

[CR82] Slepnev VI, De Camilli P (2000). Accessory factors in clathrin-dependent synaptic vesicle endocytosis. Nat Rev Neurosci.

[CR83] Stachowiak JC, Schmid EM, Ryan CJ (2012). Membrane bending by protein-protein crowding. Nat Cell Biol.

[CR84] Stachowiak JC, Brodsky FM, Miller EA (2013). A cost-benefit analysis of the physical mechanisms of membrane curvature. Nat Cell Biol.

[CR85] Steinem C, Meinecke M (2020). ENTH domain-dependent membrane remodelling. Soft Matter.

[CR86] Stoldt S, Wenzel D, Hildenbeutel M (2012). The inner-mitochondrial distribution of Oxa1 depends on the growth conditions and on the availability of substrates. Mol Biol Cell.

[CR87] Strauss M, Hofhaus G, Schroder RR, Kuhlbrandt W (2008). Dimer ribbons of ATP synthase shape the inner mitochondrial membrane. EMBO J.

[CR88] Takei K, Slepnev VI, Haucke V, De Camilli P (1999). Functional partnership between amphiphysin and dynamin in clathrin-mediated endocytosis. Nat Cell Biol.

[CR89] Tarasenko D, Barbot M, Jans DC (2017). The MICOS component Mic60 displays a conserved membrane-bending activity that is necessary for normal cristae morphology. J Cell Biol.

[CR90] Taylor MJ, Perrais D, Merrifield CJ (2011). A high precision survey of the molecular dynamics of mammalian clathrin-mediated endocytosis. PLoS Biol.

[CR91] van den Brink-van der Laan E, Killian JA, de Kruijff B (2004). Nonbilayer lipids affect peripheral and integral membrane proteins via changes in the lateral pressure profile. Biochim Biophys Acta.

[CR92] van Meer G (2005). Cellular lipidomics. EMBO J.

[CR93] van Meer G, Voelker DR, Feigenson GW (2008). Membrane lipids: where they are and how they behave. Nat Rev Mol Cell Biol.

[CR94] Verkleij AJ, Leunissen-Bijvelt J, de Kruijff B (1984). Non-bilayer structures in membrane fusion. Ciba Found Symp.

[CR95] von der Malsburg K, Müller JM, Bohnert M (2011). Dual role of mitofilin in mitochondrial membrane organization and protein biogenesis. Dev Cell.

[CR96] Williams RJ (2000). Mitochondria and chloroplasts: localized and delocalized bioenergetic transduction. Trends Biochem Sci.

[CR97] Wollert T, Hurley JH (2010). Molecular mechanism of multivesicular body biogenesis by ESCRT complexes. Nature.

[CR98] Wurm CA, Jakobs S (2006). Differential protein distributions define two sub-compartments of the mitochondrial inner membrane in yeast. FEBS Lett.

[CR99] Yoon Y, Tong J, Lee PJ (2010). Molecular basis of the potent membrane-remodeling activity of the epsin 1 N-terminal homology domain. J Biol Chem.

[CR100] Zeno WF, Baul U, Snead WT (2018). Synergy between intrinsically disordered domains and structured proteins amplifies membrane curvature sensing. Nat Commun.

[CR101] Zhang D, Zhang Y, Ma J (2020). Cryo-EM structures of S-OPA1 reveal its interactions with membrane and changes upon nucleotide binding. Elife.

[CR102] Zick M, Rabl R, Reichert AS (2009). Cristae formation-linking ultrastructure and function of mitochondria. Biochim Biophys Acta.

[CR103] Zimmerberg J, Kozlov MM (2006). How proteins produce cellular membrane curvature. Nat Rev Mol Cell Biol.

